# T cell interaction with activated endothelial cells primes for tissue-residency

**DOI:** 10.3389/fimmu.2022.827786

**Published:** 2022-09-12

**Authors:** Judith Wienke, Saskia R. Veldkamp, Eva M. Struijf, Fjodor A. Yousef Yengej, M. Marlot van der Wal, Annet van Royen-Kerkhof, Femke van Wijk

**Affiliations:** ^1^ Center for Translational Immunology, University Medical Center Utrecht, Utrecht, Netherlands; ^2^ Pediatric Rheumatology and Immunology, Wilhelmina Children’s Hospital, University Medical Center Utrecht, Utrecht, Netherlands

**Keywords:** tissue-resident memory T cells, CD69, endothelial cell, inflammation, T cell differentiation

## Abstract

Tissue-resident memory T cells (TRM) are suspected drivers of chronic inflammation, but their induction remains unclear. Since endothelial cells (EC) are obligate interaction partners for T cells trafficking into inflamed tissues, they may play a role in TRM development. Here, we used an *in vitro* co-culture system of human cytokine-activated EC and FACS-sorted T cells to study the effect of EC on T(RM) cell differentiation. T cell phenotypes were assessed by flow cytometry, including proliferation measured by CellTrace Violet dilution assay. Soluble mediators were analyzed by multiplex immunoassay. Co-culture of T cells with cytokine-activated, but not resting EC induced CD69 expression without activation (CD25, Ki67) or proliferation. The dynamic of CD69 expression induced by EC was distinct from that induced by TCR triggering, with rapid induction and stable expression over 7 days. CD69 induction by activated EC was higher in memory than naive T cells, and most pronounced in CD8^+^ effector memory T cells. Early CD69 induction was mostly mediated by IL-15, whereas later effects were also mediated by interactions with ICAM-1 and/or VCAM-1. CD69^+^ T cells displayed a phenotype associated with tissue-residency, with increased CD49a, CD103, CXCR6, PD-1 and CD57 expression, and decreased CD62L and S1PR1. EC-induced CD69^+^ T cells were poised for high production of pro-inflammatory cytokines and showed increased expression of T-helper 1 transcription factor T-bet. Our findings demonstrate that activated EC can induce functional specialization in T cells with sustained CD69 expression, increased cytokine response and a phenotypic profile reminiscent of TRM. Interaction with activated EC during transmigration into (inflamed) tissues thus contributes to TRM-residency priming.

## Introduction

Endothelial cells (EC) play a crucial role in the homeostasis of immune responses. During tissue inflammation, EC become actively engaged in response to pro-inflammatory cytokines, as is observed in a variety of inflammatory conditions such as acute graft-versus-host disease (aGvHD) and immune-mediated inflammatory diseases, including (juvenile) dermatomyositis, systemic lupus erythematosus, and scleroderma/systemic sclerosis (1–8). Especially the T cell-derived cytokines IFNγ and TNFα are important cues for EC, as they can induce the expression of costimulatory molecules and adhesion molecules (VCAM-1, ICAM-1) on EC and increase the secretion of chemo-attractants (1, 9, 10). With these, activated EC recruit leukocytes, including T cells, to inflamed tissue sites and facilitate their transmigration into tissues. This process is crucial to the surveillance and effector functions of the immune system, such as eradication of invading pathogens, but also contributes to the immunopathogenesis of inflammatory, auto- and allo-immune disorders (1, 11–13).

Transmigration of immune cells across the endothelium is a complex and slow process, which is regulated at different stages. Following priming in the lymph nodes, antigen-experienced T cells are recruited to sites of inflammation by chemo-attractants released by activated EC and tissue cells. T cells are captured from the circulation by interactions between integrins expressed by T cells, and both selectins and integrins like ICAM-1, VCAM-1 on EC. Subsequently, T cells adhere to EC and migrate through the endothelial layer into the tissue (10, 14, 15). Since the process of transmigration involves prolonged, close interaction between T cells and EC, this process might induce functional changes in transiting T cells that prepare them for the tissue environment. However, the exact changes induced by non-cognate interactions between EC and T cells are still elusive.

T cells can either transiently pass through tissues to perform their effector function and subsequently re-enter circulation, or become resident and stay in tissues for prolonged periods. These so-called tissue-resident memory T cells (TRM) play a role in tissue homeostasis, but have also gained interest because of their relevance in chronic inflammation, vaccine development, and transplantation settings (16–21). TRM are characterized by high and sustained expression of CD69, which prevents tissue egress by sequestering sphingosine-1-phosphate receptor 1 (S1PR1) from the cellular surface (22, 23). In activated T cells, short-term expression of CD69 is suggested to temporarily limit egress from lymph nodes, while constitutive expression, as found in TRM, enables long-term tissue-residency (24). Also in immune-mediated inflammatory diseases, tissue-infiltrating T cells show increased expression of CD69 and have been implicated in disease chronicity (18, 25–27). TRM are characterized by an ‘activated yet resting state’, are poised to rapidly respond to pathogens by secretion of cytokines like IFNγ, and have a decreased turnover rate compared to circulating memory cells (22, 25, 28). Furthermore, CD49a, CD103, CXCR6, CD57 and PD-1 were described as core markers for TRM, as their expression patterns best discriminate between CD69^+^ and CD69^-^ cells in different tissues (22, 29). Despite the efforts made to elucidate human TRM phenotype and function, key events in the induction of the TRM program are still poorly understood. Also the potential role of EC in the activation of T cells migrating to inflamed tissue sites is still under discussion (10).

We hypothesized that transmigration through activated endothelium into inflamed tissues may prime T cells for tissue-residency and initialize the functional specialization towards TRM. We used an *in vitro* co-culture system of human cytokine-activated EC and highly purified T cell populations to investigate the effect of EC on T cell activation and phenotype and elucidate the involved mechanisms. To mimic the microvascular EC activation involved in immune-mediated inflammatory diseases, we used a microvascular EC line.

## Materials and methods

### Endothelial cell culture

The human dermal microvascular EC line HMEC-1 (HMEC, *ATCC*) was cultured in MCDB-131 medium (*Life Technologies*) with 10 mM L-glutamine (*Gibco*), 10 ng/ml epidermal growth factor (EGF) (*Invitrogen*), 1 μg/ml hydrocortisone (*Sigma*), 1% Penicillin Streptomycin (p/s, *Gibco*) and 10% fetal calf serum (FCS) (*Biowest*). Human umbilical vein EC (HUVEC) were cultured in EGM-2 medium (Lonza) containing hEGF, hydrocortisone, GA-1000 (gentamycin, amphotericin-B), VEGF, hFGF-B, R3-IGF-1, ascorbic acid, heparin and 10% FCS. Medium was refreshed every 3-4 days and cells were suspended at confluence, using 0.05% Trypsin (*Gibco*). For phenotyping, 250.000 EC were stimulated with 10 ng/ml TNFα (*Miltenyi*) and/or 10 ng/ml IFNγ (*eBioscience*) for three days. EC were detached using 0.25 mL trypsin 0.5% EDTA *(Life Technologies)* and stained with surface antibodies for flow cytometric analysis.

### Lymphocyte isolation

Fresh peripheral blood was obtained from healthy adult volunteers, after informed consent, as approved by the Medical Ethical Committee of the University Medical Center Utrecht. Peripheral Blood Mononuclear Cells (PBMCs) were isolated by Ficoll-Paque™ PLUS (*GE Healthcare*) density centrifugation. PBMCs were frozen at -80°C until further use. CD3^+^ bulk T cells, CD3^+^ memory (CD3^+^CD45RO^+^CD45RA^-^) or naive (CD3^+^CD45RO^-^CD45RA^+^) T cells, CD8^+^ memory subsets (CD3^+^CD8^+^CD45RA^+/-^CCR7^+/-^(CD27^+/-^)) and HLA-DR^+^CD14^-^CD11c^+^ conventional dendritic cells (cDc) were obtained by fluorescence-activated cell sorting (FACS) using a FACSAria™ III cell sorter. The four CD8^+^ memory T cell subsets were defined as central memory (CM, CD45RA^-^CCR7^+^), terminally differentiated CD45RA^+^ effector memory (TEMRA, CD45RA^+^CCR7^-^) and two subsets of effector memory (EM, CD27^+^ and CD27^-^, both CD45RA^-^CCR7^-^). For proliferation assays, T cells were labeled with 2 μM Celltrace Violet (CTV, *Life Technologies*) and proliferation was assessed by flow cytometric CTV dilution assay.

### Endothelial cell-T cell co-culture

EC were plated in culture medium (RPMI 1640 with L-glutamine, p/s, and 10% FCS) in round-bottom 96-well plates (12.500 cells/well) overnight and stimulated with 10 ng/ml TNFα and/or 10 ng/ml IFNγ for three days. Afterwards, TNFα and IFNγ were removed and 50.000 FACS-sorted T cells were added per well, either with or without blocking antibodies. In control conditions, T cells were cultured in the absence of EC, in the presence of resting EC, or in the presence of 1 μg/mL soluble anti-CD3 or anti-CD3/CD28 human T-activator Dynabeads™ (1:50, *Gibco*) and/or 10.000 cDCs as indicated in the figure legends. Samples were incubated for up to 7 days and protein expression levels were analyzed using flow cytometry. Monoclonal antibodies blocking interleukin (IL)-15, transforming growth factor β (TGF-β), ICAM-1, or VCAM-1 were added to the co-culture in different concentrations (details in **Supplementary Table 1**). MHC-I interactions were blocked with an antibody against HLA-ABC (W6/32, 35 μg/mL, *Bioceros)*. To assess the effects of EC cultured medium on T cells, supernatant of cultured EC was transferred to T cells and diluted 1:1 with culture medium. Protein levels of IL-15, TGF-β, soluble ICAM-1 and soluble VCAM-1 were analyzed in undiluted EC supernatants by multiplex immunoassay, as described previously (30). To further compare the effects of soluble factors with direct cell-cell contact, a transwell co-culture assay was performed with 24-well Transwell^®^ cell culture chambers with a Polycarbonate Membrane with 0.4 μm pores (*Corning*). EC were either grown in the lower wells (75.000 cells/well) overnight (soluble factors only) or in both the lower wells (62.500 cells/well) and the upper wells (12.500 cell/well) (direct cell-cell contact and soluble factors). EC were then stimulated with TNFα and IFNγ as described above. After three days, FACS-sorted CD3^+^ memory T cells were added to the upper wells (50.000 cells/well), either with or without blocking antibodies, after removal of TNFα and IFNγ. Samples were incubated up to 7 days after which CD69 expression levels of the T cells were analyzed using flow cytometry.

### Flow cytometry

Viability of all cells was assessed with fixable viability dye eFluor505 (*eBioscience™*), by 30-minute staining at 4°C in PBS. Surface staining was performed in PBS (*Sigma)* with 2% FCS, 2% normal mouse serum (Fitzgerald) and either 0,1% NaN_3_ (*Severn Biotech Ltd.*) or 2 mM EDTA, for 20 minutes at 4°C (antibodies in **Supplementary Table 2**). For intracellular and intranuclear staining, cells were fixated and permeabilized with 1:3 Fixation/Permeabilization concentrate and Fixation/Permeabilization diluent (*Invitrogen*) for 30 minutes at 4°C. Intracellular staining was performed in permeabilization buffer (*Invitrogen*), at 4°C and for 25 minutes. Expression of cytokines was measured after four hours of restimulation with 20 ng/ml Phorbol 12-myristate 13-acelate (PMA, *Sigma*) and 1 μg/ml ionomycin (*Sigma*) in RPMI 1640 with 10% AB serum (*Sanquin*) in the presence of 1:1500 diluted GolgiStop (*BD bioscience*). Cells were subsequently rested for 90 minutes at 37°C. For optimal measurement of CD69 in PMA/ionomycin stimulated samples, CD69, CD3, CD4 and CD8 were stained prior to stimulation. Samples were measured on a BD FACSCanto™ II machine.

### Statistical analysis

Data were analyzed with FlowJo™ V10 software (*FlowJo, LLC)*. GraphPad Prism 7.02 (GraphPad Software Inc) was used for statistical analysis and graphic display of the results. For comparisons between two groups, a non-parametric T-test (Mann-Whitney U test) was used. For comparisons between more than two groups, a non-parametric ANOVA (Kruskal-Wallis test with Dunn’s *post-hoc* test) was used. For multi-level analyses (e.g. expression over time), a 2-Way-ANOVA with Sidak *post-hoc* test was used.

## Results

### Activated EC induce T cell CD69 expression, but not proliferation

**Figure 1:**
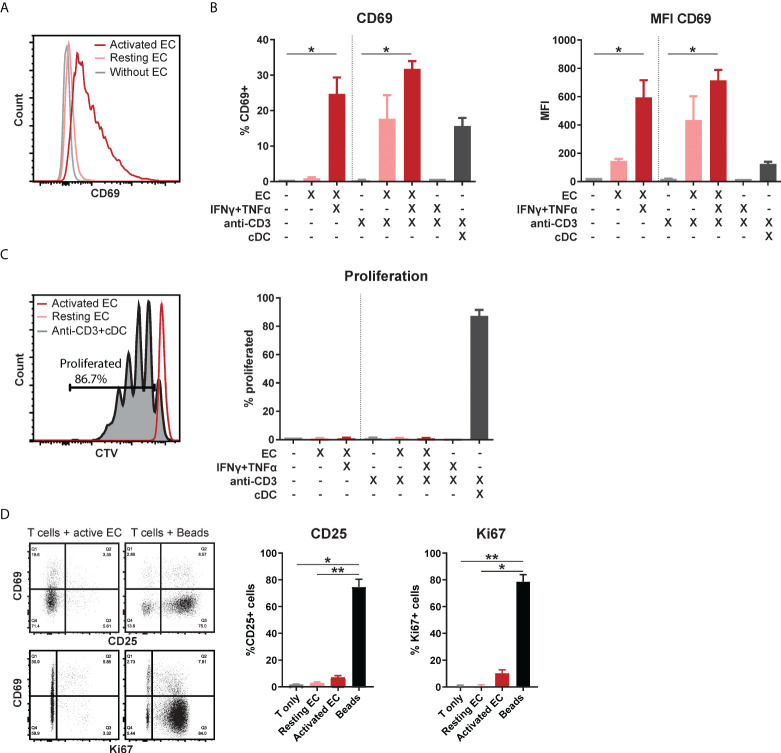
Activated EC induce CD69 expression in T cells, without proliferation or activation. HMEC were left unstimulated or stimulated with TNFα and IFNγ for 3 days before addition of FACS-sorted CD3+ T cells to the co-culture, in the presence or absence of soluble anti-CD3 stimulation. Conventional dendritic cells (cDC) were added as a positive control to induce T cell proliferation. CD69 expression was analyzed by flow cytometry after 4 days of co-culture. **(A)** Representative flow cytometry plot, **(B)** percentage of positive cells and median fluorescent intensity (MFI). Kruskal-Wallis with Dunn’s post-hoc test compared to unstimulated, c.q. only anti-CD3 stimulated T cells. *p < 0.05. **(C)** Proliferation was assessed by CellTrace Violet (CTV) dilution assay. Kruskal-Wallis with Dunn’s post-hoc test. **(D)** Expression of CD25 and Ki67 after 4 days of co-culture with unstimulated T cells, resting/activated HMEC or anti-CD3/CD28 beads. N = 3, mean+SEM. Kruskal-Wallis with Dunn’s post-hoc test. *p < 0.05, **p < 0.01.

To study the effect of the activation state of EC on T cell function, HMEC were stimulated with IFNγ and/or TNFα for 3 days. HMEC stimulated with both cytokines expressed high levels of adhesion molecules ICAM-1 and VCAM-1, HLA-DR, HLA-ABC and CD40, but negligible levels of CD80 and CD86 (**Supplementary Figure 1**). Total CD3^+^ T cells co-incubated with stimulated, but not resting HMEC, showed significantly increased expression of early activation marker CD69 compared to unstimulated T cells (**Figures 1A, B**). Additional activation of T cells by T cell receptor (TCR) stimulation with soluble anti-CD3 did not further increase CD69 expression. The combination of the cytokines TNFα and IFNγ in the absence of HMEC did not induce CD69 expression in T cells, indicating that the effect was mediated by HMEC. Although co-incubation with activated HMEC induced CD69 expression, which is usually associated with subsequent T cell proliferation (31, 32), this was not observed (**Figure 1C**). A control condition with T cells co-incubated with conventional dendritic cells (cDC) showed that the proliferative capacity of these T cells was intact. Expression of two other markers associated with T cell proliferation, Ki67 and CD25 (32), was also low in the condition with activated EC (**Figure 1D**). This indicates that activated HMEC induce CD69 expression in T cells, without inducing proliferation or conventional activation. In all follow-up experiments, T cells in co-culture with EC were not stimulated with anti-CD3.

### Activated EC induce a T cell CD69 expression dynamic distinct from conventional TCR stimulation

**Figure 2:**
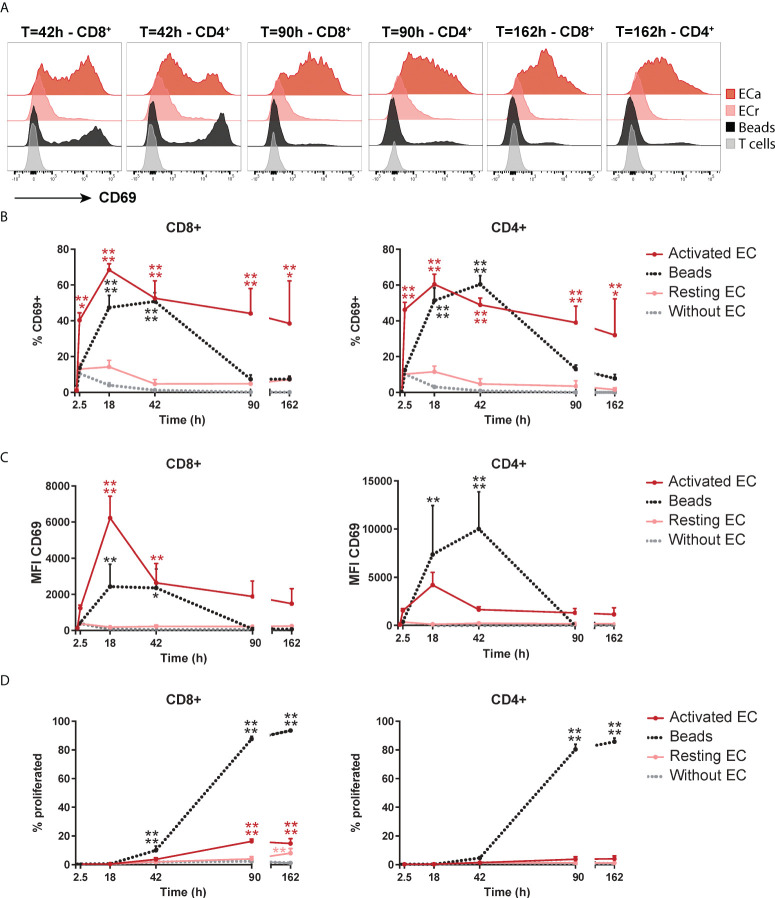
Activated EC induce a distinct dynamic of CD69 expression on T cells. HMEC were left unstimulated (resting) or stimulated with 10 ng/mL TNFα and IFNγ for 3 days (activated) before addition of FACS-sorted CD3+ T cells to the co-culture. CD69 expression and proliferation were assessed at various time points of the co-culture. As a positive control, T cells were cultured with anti-CD3/CD28 beads. **(A)** Representative flow cytometry plots of CD69 expression in CD4+ and CD8+ T cells over time. **(B)** Percentage of CD69+ cells within CD4+ and CD8+ T cells. **(C)** Median fluorescent intensity (MFI) of CD69 expression on T cells. **(D)** Percentage of proliferated T cells assessed by CellTrace Violet dilution assay. N = 3, mean+SEM. 2-Way-ANOVA with Sidak post-hoc test. *P < 0.05, **P < 0.01, ***P < 0.001, ****P < 0.0001.

To investigate the dynamics of this unusual proliferation-independent CD69 expression induced by EC, we assessed its expression over time in CD4^+^ and CD8^+^ T cells. As a control for conventional TCR stimulation-induced CD69 expression, T cells were stimulated with anti-CD3/anti-CD28 beads. HMEC-induced CD69 expression was rapid, showing an increase already after 2.5 hours, which peaked at 18 hours and maintained a marginally lower, but rather stable expression up to 7 days (162 hours) of co-culture (**Figures 2A, B**). Bead-induced CD69 expression showed a slower increase, with a peak at 18-42 hours and a sharp decline afterwards. This indicates that activated HMEC can induce rapid and sustained CD69 expression in T cells, a dynamic distinct from CD69 expression induced by TCR stimulation (33). Whereas the expression pattern of CD69 was similar in CD4^+^ and CD8^+^ T cells, the peak fluorescent intensity of CD69 induced by HMEC was higher in CD8^+^ T cells (**Figure 2C**). Remarkably, in CD8^+^ T cells HMEC induced an even higher fluorescent intensity of CD69 than beads. Again, in contrast to bead-stimulated T cells, HMEC-stimulated T cells did not proliferate (**Figure 2D**). To assess whether the capability to induce CD69 was specific to human microvascular EC or would be a general feature of EC, we repeated these experiments with HUVEC. The dynamic of CD69 expression induced by HMEC and HUVEC was essentially identical, indicating that this is a global endothelial effect (**Supplementary Figure 2**). Taken together, activated EC induce sustained CD69 expression (without proliferation) in T cells, with a dynamic distinct from CD69 expression induced by TCR stimulation.

### EC-induced CD69 expression is most pronounced in effector memory CD8^+^ T cells

**Figure 3:**
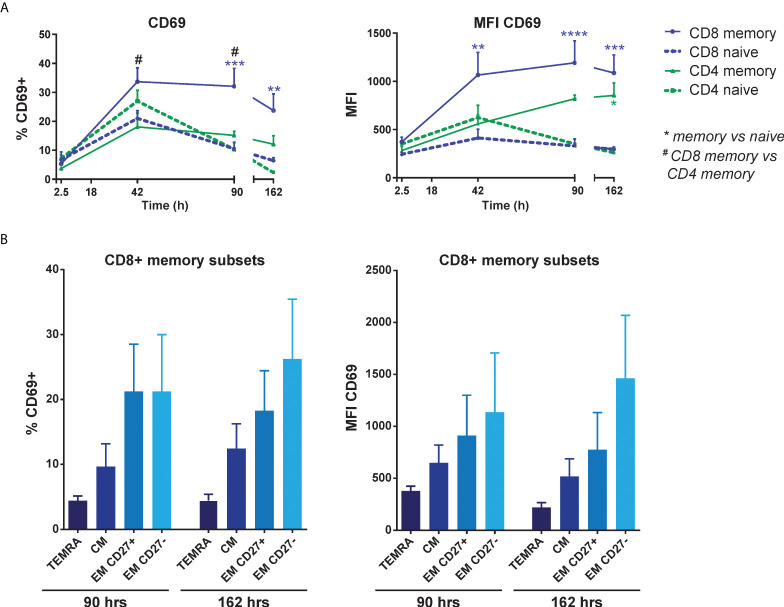
EC-induced CD69 expression is most pronounced in effector memory CD8+ T cells. HMEC were stimulated with 10 ng/mL TNFα and IFNγ for 3 days before addition of FACS-sorted naive CD3+ or memory CD3+ T cells to the co-culture. CD69 expression was assessed at various time points of the co-culture by flow cytometry. **(A)** Percentage of CD69+ cells (left panel) and median fluorescent intensity (MFI) of CD69 (right panel) on naive and memory CD4+ and CD8+ T cells. N = 4, mean+SEM. 2-Way-ANOVA with Sidak post-hoc test. */#P < 0.05, **P < 0.01, ***P < 0.001,****P < 0.0001. **(B)** Percentage of CD69+ cells (left panel) and median fluorescent intensity (MFI) of CD69 expression (right panel) on sorted CD8+ memory T cell subsets after co-culture with activated HMEC. TEMRA, terminally differentiated CD45RA+ effector memory T cells (CD45RA+CCR7-); CM, central memory T cells (CD45RA-CCR7+); EM, effector memory T cells (CD45RA-CCR7-). N = 6, mean+SEM. 2-Way-ANOVA with Sidak post-hoc test.

To assess which T cell subsets were most responsive to induction of CD69 expression, FACS-sorted naive (CD45RA^+^CD45RO^-^) and memory (CD45RA^-^CD45RO^+^) CD3^+^ T cells were separately co-cultured with HMEC. Both CD4^+^ and CD8^+^ memory T cells showed higher CD69 expression than their naive counterparts, especially after a longer culture period, and CD8^+^ memory T cells in particular had the highest and most stable expression of CD69 over time (**Figure 3A**). To identify which subpopulation(s) of CD8^+^ memory T cells were responsive to CD69 induction, we sorted 4 different subsets of CD8^+^ T cells: central memory (CM, CD45RA^-^CCR7^+^), terminally differentiated CD45RA^+^ effector memory (TEMRA, CD45RA^+^CCR7^-^) and two subsets of effector memory (EM, CD27^+^ and CD27^-^, both CD45RA^-^CCR7^-^) CD8^+^ T cells. After co-culture with activated HMEC, the two effector memory subsets, and especially the CD27^-^ subset which is associated with increased effector function (34), showed a trend of the highest and most stable CD69 expression (**Figure 3B**). These results indicate that CD8^+^ effector memory T cells are most responsive to induction and maintenance of CD69 expression by activated EC.

### EC-induced CD69 expression is partly mediated by synergistic action of IL-15, ICAM-1 and VCAM-1

**Figure 4:**
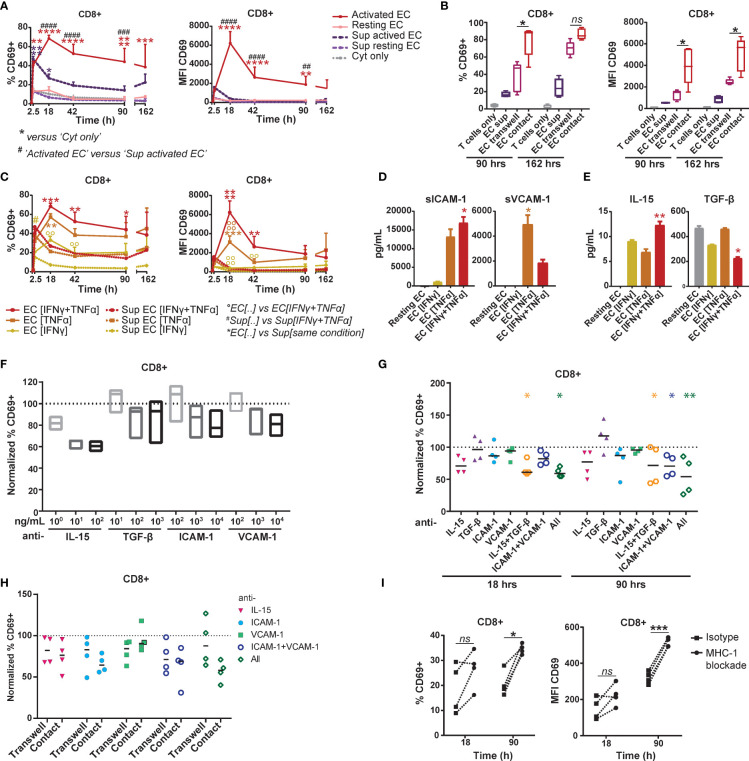
EC-induced CD69 expression on memory CD8+ T cells is partly mediated by synergistic action of IL-15, ICAM-1 and VCAM-1. (A+B+C) HMEC were left unstimulated (resting) or stimulated with 10 ng/mL TNFα and/or IFNγ for 3 days (activated) before addition of FACS-sorted memory CD3+ T cells to the co-culture or HMEC cultured medium. CD69 expression and proliferation were assessed at various time points of the co-culture. **(A)** Percentage of CD69+ cells (left panel) and median fluorescent intensity (MFI) of CD69 (right panel) within CD8+ T cells after co-culture with TNFα and IFNγ-stimulated HMEC, resting HMEC, their cultured medium (sup), or TNFα and IFNγ alone (cyt only). N=3, mean+SEM. 2-Way-ANOVA with Sidak post-hoc test. */#/^0^P < 0.05, **/##/^00^P < 0.01, ***/###/^000^P < 0.001,****/####/^0000^P < 0.0001. **(B)** Percentage of CD69+ cells (left panel) and median fluorescent intensity (MFI) of CD69 (right panel) within CD8+ T cells after culture with activated HMEC cultured medium (sup), transwell co-culture or direct co-culture with activated HMEC. N = 5, median. 2-Way ANOVA with Sidak’s multiple comparison test; *p < 0.05, ns = not significant. **(C)** Percentage of CD69+ cells (left panel) and median fluorescent intensity (MFI) of CD69 (right panel) within CD8+ T cells after co-culture with TNFα- and/or IFNγ-stimulated HMEC or their cultured medium (sup). N = 3, mean+SEM. 2-Way-ANOVA with Sidak post-hoc test. */#/^0^P < 0.05, **/##/^00^P < 0.01, ***/###/^000^P < 0.001,****/####/^0000^P < 0.0001. (D+E) Levels of soluble ICAM-1 and VCAM-1 **(D)** and IL-15 and TGF-β **(E)** measured in cultured medium of resting or TNFα- and/or IFNγ-stimulated HMEC after 3 days, by multiplex immunoassay. N = 3, mean+SEM. Kruskal-Wallis with Dunn’s post-hoc test versus resting EC. *P < 0.05, **P < 0.01. (F+G) Co-culture of TNFα and IFNγ-stimulated HMEC with FACS-sorted memory CD3+ T cells in the presence of (increasing concentrations) of monoclonal antibodies blocking IL-15, TGF-β, ICAM-1 and/or VCAM. **(F)** The percentage of CD69+ cells was measured by flow cytometry after 18 hours and normalized to the percentage of CD69+ cells in the condition with isotype control (set to 100). N = 3, median. Kruskal-Wallis with Dunn’s post-hoc test versus isotype. **(G)** The percentage of CD69+ cells was measured by flow cytometry after 18 and 90 hours and normalized to the condition with isotype control (set to 100). N = 4, median. 2-Way-ANOVA with Sidak post-hoc test versus isotype. *P < 0.05, **P < 0.01. **(H)** Transwell and direct co-culture of TNFα and IFNγ-stimulated HMEC with FACS-sorted memory CD3+ T cells in the presence of monoclonal antibodies blocking IL-15, ICAM-1 and/or VCAM. Percentage of CD69+ cells within CD8+ T cells was measured by flow cytometry after 90 hours and normalized to the condition with isotype control (set to 100). N = 4, median. 2-Way-ANOVA with Sidak post-hoc test versus isotype. **(I)** Co-culture of TNFα and IFNγ-stimulated HMEC with FACS-sorted memory CD3+ T cells in the presence of 35 μg/mL monoclonal antibody blocking HLA-ABC or isotype control. The percentage of CD69+ cells and median fluorescent intensity (MFI) of CD69 was measured by flow cytometry after 18 and 90 hours. N = 4. Dotted lines indicate paired measurements. 2-Way-ANOVA with Sidak post-hoc test versus isotype. *P < 0.05, ***P < 0.001.

To elucidate the mechanism behind EC-mediated induction of CD69, we separated effects mediated by cell-contact and soluble factors by culturing T cells in the direct presence of HMEC or their cultured medium. HMEC culture supernatants induced a rapid, but lower CD69 expression on CD8^+^ T cells than direct co-culture with HMEC (**Figure 4A**). Supernatant-induced CD69 expression was also less stable, possibly due to consumption of soluble factors. We therefore validated the contribution of soluble factors to CD69 expression in a transwell co-culture system (**Figure 4B**). Although HMEC-derived soluble factors induced a substantial CD69 expression in the transwell co-culture, CD69 expression induced by direct contact was significantly higher. This indicated that soluble factors contributed to, but were not solely responsible for CD69 induction, thereby attributing an important role to direct cell-contact.

HMEC showed differential upregulation of adhesion and costimulatory molecules in response to stimulation with IFNγ and TNFα (**Supplementary Figure 1**). To identify candidate molecules which could mediate CD69 induction by cell-contact or in solution, we analyzed the differential effect of IFNγ- and/or TNFα-stimulated HMEC on CD69 expression by T cells. Both direct co-culture and supernatant of TNFα-stimulated HMEC induced higher levels of CD69 than IFNγ-stimulated HMEC (**Figure 4C**), indicating that molecules upregulated by HMEC upon TNFα stimulation contributed most to CD69 induction. HMEC stimulated with both cytokines effected only slightly more CD69 expression than HMEC stimulated with only TNFα, which suggested that the IFNγ-mediated effect was small. Expression of ICAM-1 and VCAM-1 on HMEC was most dependent on TNFα stimulation, thereby mirroring the identified pattern of CD69 induction, which rendered them plausible candidate molecules. Soluble levels of ICAM-1 and VCAM-1 as measured in culture supernatants of activated HMEC were also induced by TNFα stimulation (**Figure 4D**).

Two soluble factors, IL-15 and TGF-β, have been previously shown to increase CD69 expression on T cells (35–37). In culture supernatant of activated HMEC IL-15 production was similarly induced by IFNγ and TNFα stimulation, whereas TGF-β appeared to be constitutively produced and downregulated by IFNγ stimulation (**Figure 4E**). Although these patterns did not match the preferential pattern of CD69 induction by TNFα-stimulated HMEC, we empirically blocked their actions in memory T cell-HMEC co-cultures, as well as that of ICAM-1 and VCAM-1. Expression of their respective receptors on T cells is shown in **Supplementary Figure 3A**. Respective blockade of IL-15, ICAM-1 and VCAM-1 resulted in a dose-dependent trend of reduction of CD69 expression, indicating that these factors are likely involved in HMEC-mediated CD69 induction (**Figure 4F**). Blockade of IL-15 reduced CD69 induction on T cells by up to 40-45% in the early phase of culture (**Figures 4F, G**). Blockade of ICAM-1 also caused a small reduction in CD69 expression, whereas blockade of TGF-β and VCAM-1 had no effect. Combined blockade of IL-15 and TGF-β contributed most to early suppression of CD69 expression on T cells (**Figure 4G**). After 4 days of co-culture, IL-15 blockade also reduced CD69 induction up to 40%, but blockade of TGF-β rather increased than decreased CD69 expression. Combined blockade of these two cytokines significantly reduced CD69 expression with an effect size similar to blockade of IL-15 alone. Blockade of ICAM-1 or VCAM-1 alone caused a non-significant reduction in CD69 expression. However, combined blockade of ICAM-1 and VCAM-1 showed a synergistic effect after 4 days of co-culture, reducing CD69 expression by up to 50%. The combined blockade of all 4 molecules caused a further reduction of CD69 expression to up to 65% on day 4. This indicates that likely a multitude of signals provided by activated EC induces CD69 expression in T cells, and that it is partly mediated by the synergistic action of IL-15, ICAM-1 and VCAM-1. To elucidate whether the soluble or membrane-bound forms of these molecules contributed most to CD69 induction, we compared blockade of IL-15, ICAM-1 and/or VCAM-1 in a transwell setting with direct co-cultures. The effect of IL-15 blockade was similar in both settings, and, although we did not observe significant differences, the effect of ICAM-1 blockade appeared larger in the direct co-culture (**Figure 4H**). Blockade of all three molecules also had the largest impact in direct co-cultures. This indicates that both soluble IL-15 and cell-bound ICAM-1 appear to contribute to CD69 induction, whereas the role of (cell-bound or soluble) VCAM-1 is less evident. We observed similar effects in CD4^+^ T cells, suggesting that they respond to similar signals provided by EC (**Supplementary Figures 3B–F**). Lastly, to test whether MHC-I dependent allogeneic recognition of EC by T cells contributed to CD69 expression, we co-cultured T cells and HMEC in the presence of an MHC-I blocking antibody (**Figure 4I**). Blockade of MHC-I-TCR interactions did not reduce CD69 expression. This indicates that MHC-I-TCR interaction is not required for CD69 induction and that most observed effects are mediated by non-cognate interactions with EC. This was further supported by the superior effect of TNFα-stimulated HMEC over IFNγ-stimulated HMEC, even though IFNγ more potently induced MHC expression.

### T cell – EC interaction as a priming signal for tissue-residency

**Figure 5:**
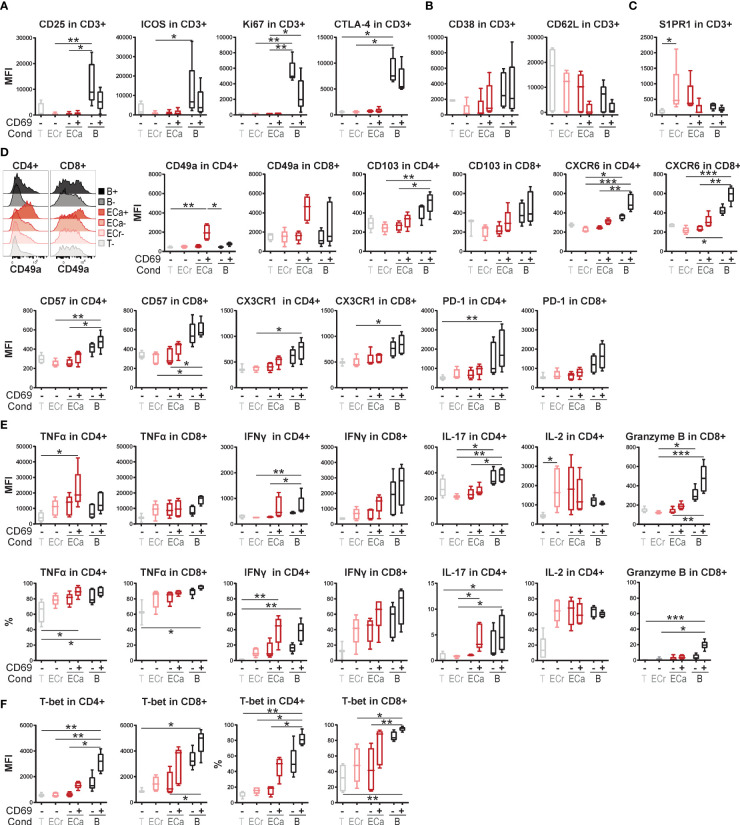
Expression of tissue-resident memory T cell associated markers in EC-stimulated T cells. **(A–F)** Co-culture of resting or TNFα and IFNγ-stimulated HMEC or anti-CD3/CD28 beads with FACS-sorted memory CD3+ T cells. Expression of activation markers **(A)** and effector/memory markers **(B)** was assessed by flow cytometry after 4 days of co-culture. Expression of markers associated with tissue-residency (**C**+**D**), cytokines **(E)** and transcription factors **(F)** was assessed by flow cytometry after 7 days of co-culture. Cytokine expression was measured intracellularly after restimulation. N = 5, boxplots with median. Cond, condition; T, T cells only (CD69-); ECr, resting EC (CD69-); ECa, activated EC (CD69- and CD69+); B, anti-CD3/CD28 beads (CD69- and CD69+). Kruskal-Wallis with Dunn’s post-hoc test. *P < 0.05, **P < 0.01,***P < 0.0001.

To investigate whether proliferation-independent CD69 expression induced by interaction with activated EC may represent one of the first signs of T cells adopting a specialized program that primes them for prolonged residency in tissues, we analyzed the co-expression of TRM-associated markers with CD69. As previously shown, after 4 days of co-culture expression levels of activation markers CD25 and Ki67, but also ICOS and CTLA-4, were significantly lower in HMEC-stimulated than in bead-activated T cells and not increased compared to T cells cultured without HMEC (**Figure 5A**), again indicating that CD69 expression in these cells does not represent conventional activation. Memory/effector marker CD38 was marginally but not significantly higher in CD69^+^ than CD69^-^ T cells cultured with activated HMEC, but lower than in bead-activated cells (**Figure 5B**). Expression levels of CD62L showed a trend of specific downregulation in CD69^+^ compared to CD69^-^ cells, indicating specialization towards an effector phenotype.

Markers associated with tissue-residency were assessed after 7 days of co-culture. S1PR1 was upregulated in CD69^-^ cells in response to co-culture with HMEC, but downregulated in CD69^+^ cells, as also described for TRM (**Figure 5C**) **(** 22, 23). Remarkably, the TRM-associated marker CD49a (ITGAM1) showed specific upregulation in CD69^+^ cells co-cultured with HMEC, but not beads, both in CD4^+^ and CD8^+^ T cells (**Figure 5D**). Other integrins CD49b and CD49d were not upregulated, indicating that EC specifically induce expression of integrin CD49a (**Supplementary Figure 4**). Expression of other TRM-related markers CD103 (ITGAE), CXCR6, CD57, CX3CR1 and PD-1 all showed a trend of higher expression in CD69^+^ compared to CD69^-^ T cells co-cultured with HMEC, but lower than in T cells stimulated with beads.

Intracellular cytokine expression was assessed after 7 days of co-culture and re-stimulation. T cells co-cultured with activated HMEC were poised for production of pro-inflammatory cytokines TNFα and IFNγ (**Figure 5E**). We observed high expression of TNFα specifically in CD4^+^CD69^+^ cells co-cultured with HMEC, which was even higher than in bead-stimulated CD4^+^ T cells, and expressed in 80-100% of CD4^+^ cells. IFNγ expression was (non-significantly) higher in CD69^+^ than CD69^-^ T cells, and comparable between HMEC-stimulated and bead-stimulated T cells. IL-17 expression was increased in CD69^+^ cells compared to CD69^-^ CD4^+^ T cells, whereas IL-2 expression was induced by HMEC and beads irrespective of CD69 expression. Absence of granzyme B expression indicated that the high cytokine response of CD69^+^ T cells co-cultured with HMEC was likely not reflective of direct cytotoxicity towards HMEC. Increased expression of Th1-related transcription factor T-bet in 25-60% of CD4^+^CD69^+^ and 45-95% of CD8^+^CD69^+^ T cells was consistent with increased IFNγ-production in CD69^+^ cells (**Figure 5F**).

Taken together, CD69^+^ T cells induced by activated HMEC do not appear to be conventionally activated, but rather express TRM-associated markers at higher levels than their CD69^-^ counterparts, and specialize into Th1-like effector memory T cells with an increased pro-inflammatory cytokine response upon stimulation.

## Discussion

Although the TRM phenotype and function in human tissues have been extensively investigated, the process of TRM induction remains a major outstanding question in the field (22, 24, 25, 38). Here, we have demonstrated that activated EC can induce sustained CD69 expression on T cells in the absence of TCR stimulation, without inducing proliferation or activation. The dynamic of this sustained CD69 expression was clearly distinct from TCR-dependent T cell activation, and EC-mediated induction of CD69 expression was partly dependent on IL-15, VCAM-1 and ICAM-1. Moreover, EC-induced CD69^+^ T cells expressed multiple markers associated with tissue-residency in T cells and were poised for a pro-inflammatory cytokine response. Our observations are consistent with the described “activated yet resting” functional phenotype of TRM (28). Therefore, close interaction with EC during transmigration appears to be one of the first cues priming tissue-infiltrating T cells for tissue-residency.

Interaction with EC, and especially transmigration, can influence T cell function and even induce proliferation in the presence of TCR stimulation during co-culture (39–41). Only a few studies have demonstrated the induction of CD69 on T cells by activated EC without TCR triggering, but this was linked to activation and not to potential TRM programming at that time (35, 42, 43). In line with our results, these previous studies showed that CD69 expression was dependent on LFA-1/ICAM-1 interaction and enhanced in the presence of IL-15 (35, 43). IL-15 can induce CD69 expression and proliferation in naive and memory T cells, but was also shown to be a crucial factor for TRM development (29, 44, 45). Based on these previous results combined with our own data, including the downregulation of S1PR1 which limits egress from tissues, we would like to propose an alternative hypothesis in which interaction of effector memory T cells with activated EC primes T cells for tissue-residency. This is also in line with published data showing that EC-T cell interaction enhances T cell responsiveness to antigenic challenge and increases T cell motility, features required for and conducive to TRM fate (43). In addition, transmigration has been shown to increase T cell survival in an ICAM-1 dependent manner, which could partly prepare them for the longevity of TRM in tissues (46, 47).

As tissue environments provide specific cues regulating functional characteristics of immune cells, TRM may represent a plastic T cell population, and both CD4^+^ and CD8^+^ TRMs may have the ability to up- and downregulate described TRM markers depending on the microenvironmental cues present at their specific tissue site (24, 38, 48, 49). The context of the tissue environment may therefore support a two-step model for development of TRM: first, interaction with or transmigration through EC primes T cells for increased receptivity towards environmental signals and increased migratory capacity, inducing an “activated yet resting” state and CD69 upregulation preventing tissue egress. Second, microenvironmental signals from the tissue environment further shape, support and consolidate the specific TRM profile that is ‘required’ at a certain tissue site. This hypothesis for TRM development is consistent with a previously suggested model of T cell trafficking, which also emphasizes a role for EC shaping T cell function (50, 51).

In steady-state conditions, TRM provide rapid on-site immune protection against known infectious pathogens. However, aberrantly activated or autoreactive TRM can contribute to the pathogenesis of chronic inflammatory diseases (18). Indeed, pathogenic TRM have been implicated in the initiation and/or relapsing course of psoriasis, Crohn’s disease and rheumatoid arthritis (18, 52). In the context of allogeneic hematopoietic stem cell transplantation (HCT), cytotoxic tissue-infiltrating donor T cells have also been demonstrated to acquire TRM features during gastrointestinal aGvHD in animal models, driving tissue destruction (21, 53). Interestingly, transcriptional analysis of these actively infiltrating cells revealed a clinically relevant gene signature associated with adhesion, extravasation and migration (21), underlining the role of the endothelium as interaction partner. Our data are further supported by newly emerging, promising therapeutic strategies targeting endothelial activation to prevent complications post-HCT (54–56). Defibrotide, for instance, a drug currently approved for hepatic veno-occlusive disease after HCT, has been shown to protect EC from pro-inflammatory activation in *in vitro* HCT models, and reduced the risk and severity of aGVHD in a large pediatric prospective randomized trial (54–56).

In our *in vitro* experimental setting with allogeneic EC, we cannot rule out that direct allorecognition of EC by T cells may have accounted for some of the observed effects. However, direct allorecognition accounting for CD69 expression is unlikely, due to the lack of induction of CD25 expression, proliferation and the absence of effect of the MHC-I blockade, as also observed previously (10, 35, 39, 57). Since we studied the effects of EC on T cell phenotype and function only in an *in vitro* system, it would be important to further investigate the hypothesis concerning priming for tissue-residency in more detail, if possible *in vivo*, to also take into account the effect of a tissue-environment.

In conclusion, we have constructed an *in vitro* system using T cells and cytokine-activated EC, with which we recapitulated the peculiar phenotypical and functional characteristics of TRM. These included sustained expression of CD69 and markers of tissue residency, as well as an “activated yet resting” state poised for rapid cytokine production. These findings support our hypothesis that interaction with EC may be one of the first events priming transmigrating T cells for the specific functional requirements of tissue-residency.

## Data availability statement

The raw data supporting the conclusions of this article will be made available by the authors, without undue reservation.

## Ethics statement

The studies involving human participants were reviewed and approved by Medical Ethical Committee (METC) Utrecht. The patients/participants provided their written informed consent to participate in this study.

## Author contributions

JW conceived and designed the study, performed experiments, analyzed data, and wrote the manuscript. SRV performed experiments, analyzed data, and wrote the manuscript. ES, FYY and MMW designed and performed experiments, and critically revised the manuscript. AR-K supervised JW and SV, critically revised the manuscript and contributed to discussions. FW conceived and supervised the overall study, critically revised the manuscript and contributed to discussions. FW had full access to all the data in the study and takes responsibility for the integrity of the data and the accuracy of the data analysis. All authors contributed to the article and approved the submitted version.

## Funding

Part of this research was funded by Cure JM.

## Acknowledgments

We would like to thank the Luminex facility for their help with measuring cytokine levels in culture supernatants.

## Conflict of interest

The authors declare that the research was conducted in the absence of any commercial or financial relationships that could be construed as a potential conflict of interest.

## Publisher’s note

All claims expressed in this article are solely those of the authors and do not necessarily represent those of their affiliated organizations, or those of the publisher, the editors and the reviewers. Any product that may be evaluated in this article, or claim that may be made by its manufacturer, is not guaranteed or endorsed by the publisher.
