# Supplementary material for: T cell interaction with activated endothelial cells primes for tissue-residency
Source: Front Immunol. 2022 Sep 12;13:827786. doi: 10.3389/fimmu.2022.827786 (PMC9510578; doi:10.3389/fimmu.2022.827786)
Supplement: Supplementary file 1 [file DataSheet_1.pdf]

SUPPLEMENTARY MATERIAL

Supplementary table 1. Blocking antibodies used in the co-culture experiments.

Antigen	Company	Clone
**IL-15**	eBioscience™	ct2nu
**TGF-β**	Biolegend	19D8
**VCAM-1**	R&D systems	BBIG-V1
**ICAM-1**	Biolegend	HCD54
**IgG1 κ Isotype control**	eBioscience™	P3.6.2.8.1
**HLA-ABC**	Bioceros BV	W6/32
**IgG2a κ Isotype control**	eBioscience™	eBM2a

Supplementary table 2. Antibodies used for flow cytometry.

Antigen	Fluorescent label	Dilution	Company	Clone
**CCR7**	APC	50x	Sony Biotechnology	G043H7
**CD3**	FITC	50×	Sony Biotechnology	OKT3
**CD3**	eF450	200×	eBioscience™	OKT3
**CD3**	PE	50x	BD Bioscience	SK7
**CD3**	V500	50x	BD Bioscience	SP34-2
**CD4**	PerCP Cy5.5	400×	Sony Biotechnology	RPA-T4
**CD8**	APC	1600×	BD Bioscience	SK1
**CD8**	APC-Cy7	50×	BD Bioscience	SK1
**CD11c**	V450	200x	BD Bioscience	B-LY6
**CD14**	APC-Cy7	100x	BD Bioscience	MphiP9
**CD25**	PE-Cy7	25×	BD Bioscience	M-A251
**CD27**	PerCP	200x	Biolegend	O323
**CD38**	PE-Cy7	50×	eBioscience™	HIT2
**CD40**	FITC	50x	eBioscience™	5C3
**CD45RO**	PB	50×	Sony Biotechnology	UCHL1
**CD45RA**	PB	200x	Biolegend	HI100
**CD45RA**	FITC	50×	BD Bioscience	HI100
**CD45RA**	APC-Cy7	400×	Sony Biotechnology	HI100
**CD49a**	AF647	1600×	Sony Biotechnology	ts2/7
**CD57**	FITC	100×	Biolegend	HNK-1
**CD62L**	APC-Cy7	50×	Biolegend	DREG-56
**CD69**	PE	25×	Miltenyi	FN50
**CD69**	PE-Cy7	50×	BD Bioscience	FN50
**CD80**	APC-H7	50x	BD Bioscience	L307.4
**CD86**	PB	50x	Biolegend	IT2.2

**CD103**	FITC	25×	Beckman Coulter	2G5
**CTLA-4**	APC	12,5×	BD Bioscience	BNI3
**CXCR6**	PE	50×	R&D systems	56811
**CX3CR1**	FITC	50×	Biolegend	2A9-1
**Granzyme B**	FITC	50×	BD Bioscience	GB11
**HLA-ABC**	APC-Cy7	100x	Sony Biotechnology	W6/32
**HLA-DR**	PerCP-Cy5.5	200x	Biolegend	L243
**HLA-DR**	PE-Cy7	400x	Biolegend	L243
**HLA-DR**	Pacific Blue	200x	Sony Biotechnology	L243
**ICAM-1**	APC	100x	BD Bioscience	HA58
**ICOS**	APC	20×	eBioscience™	ISA3
**IFN-γ**	FITC	50×	BD Bioscience	25723.11
**IL-2**	FITC	100×	BD Bioscience	MQ1-17H12
**IL-17a**	PE	100×	eBioscience™	eBio64DEC1
**Ki67**	FITC	100×	BD Bioscience	B56
**PD-1**	PE	25×	BD Bioscience	EH12.1
**S1PR1**	eF660	200×	eBioscience™	SW4GYPP
**T-Bet**	PE	50×	eBioscience™	eBio4B10
**TNF-α**	APC	400×	Biolegend	Mab11
**VCAM-1**	PE	100x	eBioscience™	STA

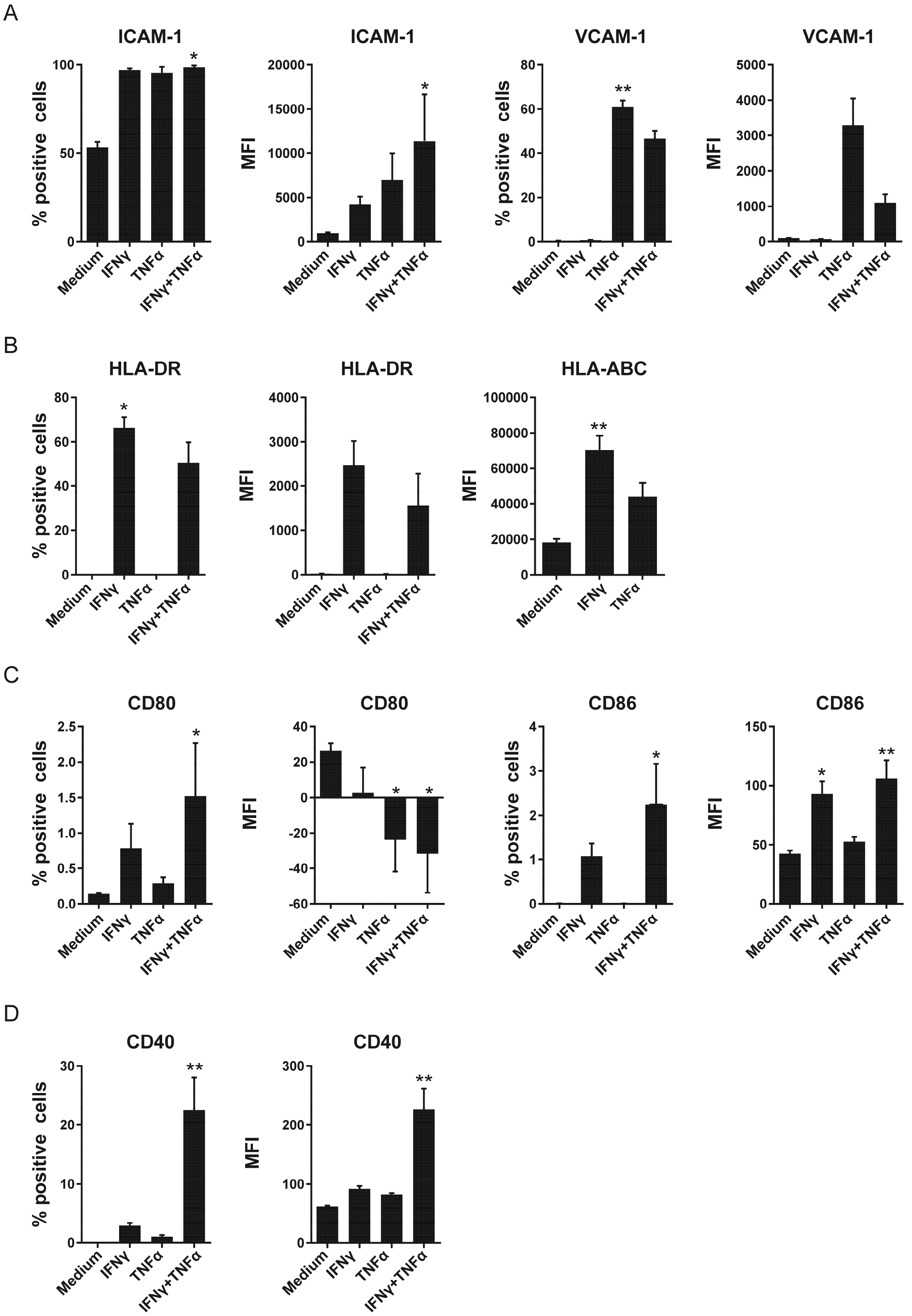

Supplementary figure 1. Expression of adhesion molecules, MHC molecules and costimulatory molecules on EC in response to stimulation with TNFα and/or IFNγ. HMEC were incubated with 10 ng/mL TNFα and/or IFNγ for 3 days. Expression of **(A)** adhesion, **(B)** MHC, and **(C+D)** costimulatory molecules was analyzed by flow cytometry. *N=4; mean+SEM. Kruskall-Wallis with Dunn's post-hoc test. *P<0.05, **P<0.01.*

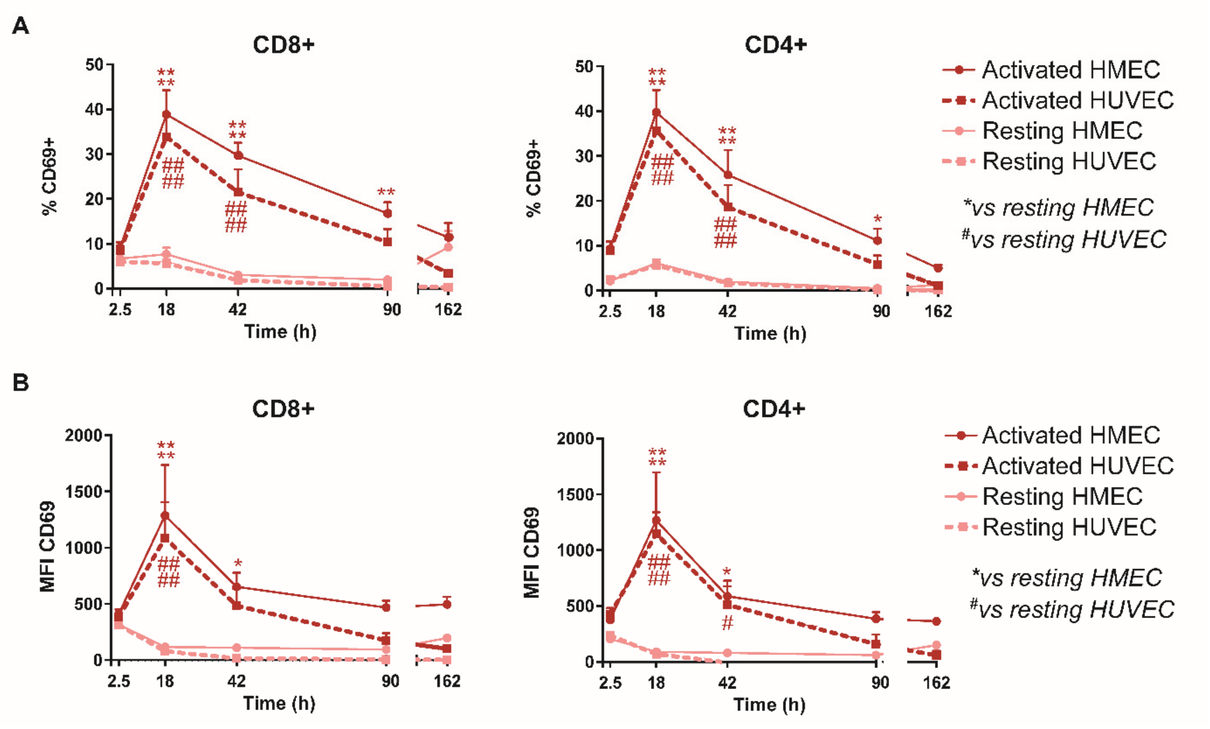

Supplementary figure 2. Dynamic of CD69 induction on T cells by HMEC and HUVEC. HMEC and HUVEC were left unstimulated (resting) or stimulated with 10 ng/mL TNFα and IFNγ for 3 days before addition of FACS-sorted $CD3^+$ T cells to the co-culture. CD69 expression on $CD8^+$ and $CD4^+$ T cells was analyzed by flow cytometry at different time points. **(A)** Percentage of $CD69^+$ cells among T cells. **(B)** Median fluorescent intensity (MFI) of CD69 expression on T cells. *N=4, mean+SEM. 2-Way-ANOVA with Sidak post-hoc test versus isotype.* $^{*/\#}P<0.05$, $^{**/\#\#}P<0.01$, $^{***/\#\#\#}P<0.001$, $^{****/\#\#\#\#}P<0.0001$.

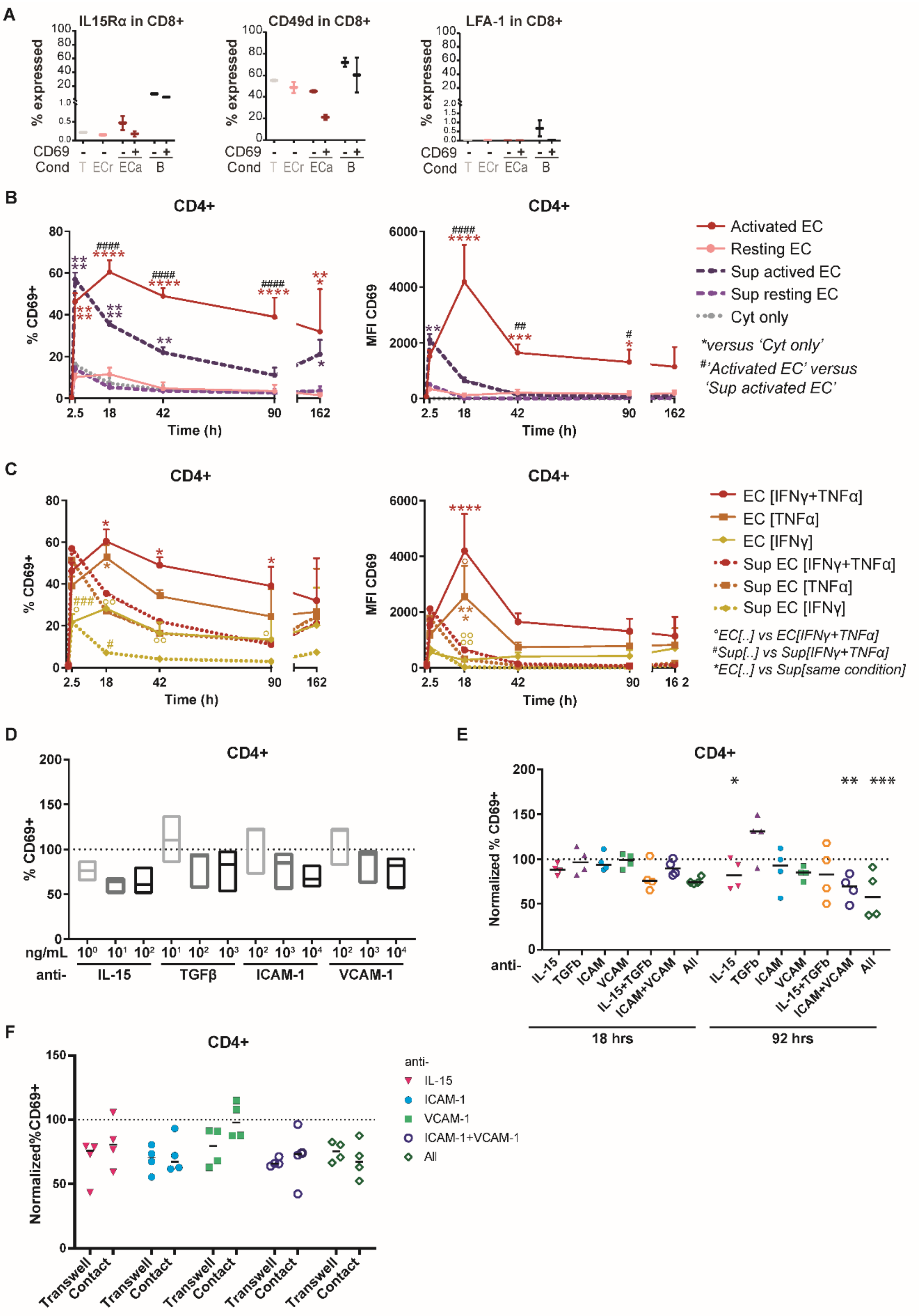

Supplementary figure 3. EC-induced CD69 expression on CD4^{+} T cells is partly mediated by synergistic action of IL-15, ICAM-1 and VCAM-1.

Supplementary figure 3 continued. (A) Expression of IL-15Rα, CD49d and LFA-1 on T cells co-cultured with resting or activated EC after 4 days, measured by flow cytometry. **(B+C)** EC were left unstimulated (resting) or stimulated with 10 ng/mL TNFα and/or IFNγ for 3 days (activated) before addition of FACS-sorted $CD3^+$ T cells to the co-culture or EC cultured medium. CD69 expression and proliferation were assessed at various time points of the co-culture. *2-Way-ANOVA with Sidak post-hoc test versus isotype.* $^{*/\#/o}P<0.05$, $^{**/\#\#/oo}P<0.01$, $^{***/\#\#\#/ooo}P<0.001$, $^{****/\#\#\#\#/oooo}P<0.0001$. **(B)** Percentage of $CD69^+$ cells (left panel) and median fluorescent intensity (MFI, right panel) of CD69 within $CD8^+$ T cells after co-culture with TNFα and IFNγ-stimulated EC, their cultured medium (sup=supernatant), or TNFα and IFNγ alone. *N=3, mean+SEM.* **(C)** Percentage of $CD69^+$ cells (left panel) and median fluorescent intensity (MFI, right panel) of CD69 within $CD8^+$ T cells after co-culture with TNFα- and/or IFNγ-stimulated EC or their cultured medium (sup). *N=3, mean+SEM.* **(D+E)** Co-culture of TNFα and IFNγ-stimulated EC with FACS-sorted memory $CD3^+$ T cells in the presence of increasing concentrations of monoclonal antibodies blocking IL-15, TGF-β, ICAM-1 and/or VCAM. **(D)** The percentage of $CD69^+$ cells was measured by flow cytometry after 18 hours and normalized to the percentage of $CD69^+$ cells in the condition with isotype control (set to 100). *N=3, median. Kruskall-Wallis with Dunn's post-hoc test versus isotype.* **(E)** The percentage of $CD69^+$ cells was measured by flow cytometry after 18 and 90 hours and normalized to the condition with isotype control (set to 100). *N=4, median. 2-Way-ANOVA with Sidak post-hoc test versus isotype. *P<0.05, **P<0.01, ***P<0.001.*

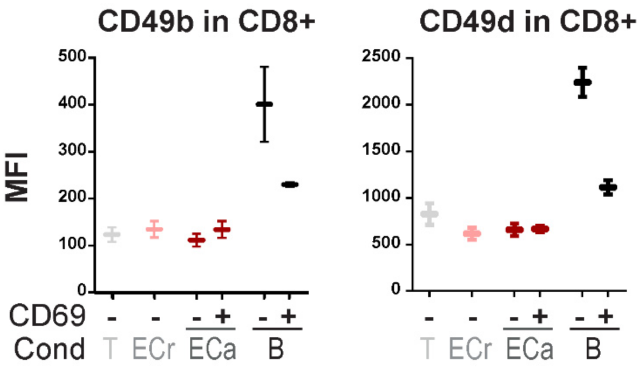

Supplementary figure 4. Expression of integrins CD49b and CD49d in EC-stimulated CD8+ T cells. Co-culture of resting or TNFα and IFNγ-stimulated HMEC or anti-CD3/CD28 beads with FACS-sorted memory $CD3^+$ T cells. Expression of integrins was assessed by flow cytometry after 7 days of co-culture. *N=3, boxplots with median. Cond = condition, T = T cells only ($CD69^-$), ECr = resting EC ($CD69^-$), ECa = activated EC ($CD69^-$ and $CD69^+$), B = anti-CD3/CD28 beads ($CD69^-$ and $CD69^+$). Kruskall-Wallis with Dunn's post-hoc test. N=2.*
